# P-102. Changes in immunologic parameters following the addition of fostemsavir in virally suppressed immune non-responders living with HIV-the RECOVER study

**DOI:** 10.1093/ofid/ofaf695.331

**Published:** 2026-01-11

**Authors:** Charlotte-Paige M Rolle, Jamie Castano, Vu Nguyen, Federico Hinestrosa, Edwin DeJesus

**Affiliations:** Orlando Immunology Center; Emory Rollins School of Public Health, Orlando, FL; Orlando Immunology Center, Orlando, Florida; Orlando Immunology Center, Orlando, Florida; Orlando Immunology Center, University of Central Florida College of Medicine, Orlando, FL; Orlando Immunology Center, University of Central Florida College of Medicine, Orlando, FL

## Abstract

**Background:**

Fostemsavir (FTR) is a pre-attachment inhibitor that binds directly to viral gp120 preventing HIV interaction with the host immune cell. This unique mechanism may drive the robust CD4^+^ T-cell recovery seen in the BRIGHTE study. Here, we evaluate changes in immunologic parameters following addition of FTR to antiretroviral (ARV) regimens among virally suppressed immune non-responders (INRs) through 48 weeks.

**Figure:**
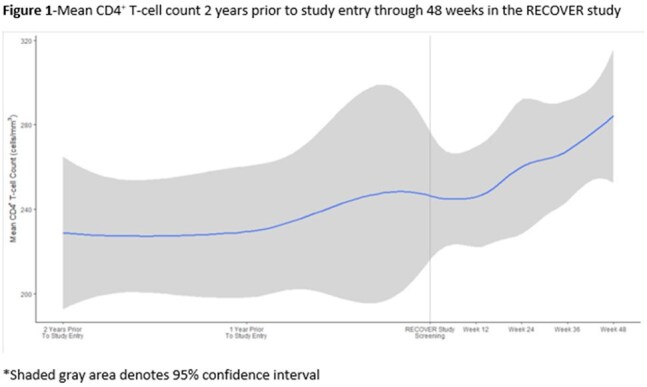

**Methods:**

RECOVER (NCT05220358) is an ongoing, open-label clinical trial that enrolled adult INRs defined as those with HIV-1 RNA<50 copies/mL and CD4^+^ T-cell count< 350 cells/mm^3^ while on suppressive ARVs for ≥2 years. Participants added FTR to their baseline ARV regimen and were followed for 48 weeks. Co-primary endpoints are mean and median change in CD4^+^ T-cell count at Week 48 using pre-FTR mean and median CD4^+^ T-cell counts 96 weeks prior to study entry as controls. 48-week data from the first 23 participants are included in this interim analysis.

**Results:**

22% enrolled were women, 74% were aged≥50 years and 65% were non-white. At baseline, mean duration of HIV infection, ARV therapy and viral suppression were 24.5, 24.7, and 18.4 years respectively. Baseline mean and median CD4^+^ T-cell counts were 248 cells/mm^3^ and 252 cells/mm^3^. At Week 48, mean and median CD4^+^ T-cell count significantly increased by 40 cells/mm^3^ and 32 cells/mm^3^ compared to pre-FTR controls (p=0.021 for mean and 0.024 for median). This was accompanied by an increase in mean and median CD4^+^ percentage of 0.5% and 0.9% compared to pre-FTR controls (p=0.372 for mean and 0.176 for median). Mean change from baseline in CD4/CD8 ratio was +0.02 (p=0.12). At Week 48, the proportion with CD4^+^ T-cell count≥350 cells/mm^3^ was 24%. Through Week 48, 2 participants had confirmed HIV-1 RNA≥50 copies/mL, however resuppressed on FTR plus the baseline ARV regimen. Drug-related adverse events (AEs) occurred in 17% and led to discontinuation in 4%. There were no drug-related serious AEs.

**Conclusion:**

Data from this interim analysis demonstrates significant increases in CD4^+^ T-cell parameters after the addition of FTR among virally suppressed INRs through Week 48. Further studies in larger samples are needed to confirm these findings and understand the biological mechanisms which may mediate these effects.

**Disclosures:**

Charlotte-Paige M. Rolle, MD, MPH, Gilead Sciences: Grant/Research Support|Gilead Sciences: Honoraria|MSD: Grant/Research Support|ViiV Healthcare: Advisor/Consultant|ViiV Healthcare: Grant/Research Support|ViiV Healthcare: Honoraria Federico Hinestrosa, MD, Gilead Sciences: Honoraria|MSD: Honoraria

